# Discovering common and population-specific QTLs for leaf rust resistance in different Barley populations

**DOI:** 10.1007/s00122-026-05375-7

**Published:** 2026-09-14

**Authors:** Cathrine Kiel Skovbjerg, Khalid Mahmood, Pernille Sarup, Jihad Orabi, Ellen Margrethe Wahlström, Jens Due Jensen, Lotte Olesen, Just Jensen, Ahmed Jahoor, Guillaume Ramstein

**Affiliations:** 1 Nordic Seed A/S, Kornmarken 1, 8464 Galten, Denmark; 2 Center for Quantitative Genetics and Genomics, Aarhus University, C.F. Møllers Allé 3, 8000, Aarhus C, Denmark

## Abstract

**Key message:**

Multi-population GWAS lead to identification of common and population-specific QTLs for leaf rust resistance in barley.

**Abstract:**

Genome-wide association studies (GWAS) are a powerful tool for detecting genetic markers associated with traits of interest. However, these studies are typically restricted to a single population, and transferability of identified marker effects across populations is challenged by population differences in linkage, allele frequencies, epistatic effects, and environmental context. When comparing GWAS results between populations, a lack of overlapping signals is often interpreted as a lack of common quantitative trait loci (QTLs), although such discrepancies may result from differences in statistical power to detect signals. In barley (*Hordeum vulgare* L.), where genetic leaf rust resistance is rapidly overcome by evolving pathogens, identification of cross-population robust and potentially transferable resistance loci is a key task. Here, we present a mixed model approach for multi-population GWAS that estimates correlated marker effects in multiple populations and use this to test for significant effects across and within populations. Applying this model to four barley breeding populations revealed both common and population-specific QTL effects for leaf rust resistance, including loci colocalizing with known *Rph* genes and novel regions with plausible candidate genes. Multi-population GWAS increased power, revealing signals not detected by GWAS within populations. We categorized the reported QTLs into three groups based on marker-associated allele effects: (1) consistent effect direction across populations, (2) differing effect direction across populations, and (3) present in a single population. The study highlights the transferability and limitations of leaf rust resistance QTLs across different barley populations and provides a general statistical framework to support robust marker-assisted selection across populations.

**Supplementary Information:**

The online version contains supplementary material available at https://doi.org/10.1007/s00122-026-05375-7.

## Introduction

Leaf rust, caused by the fungal pathogen *Puccinia hordei*, is a geographically widespread disease in barley (*Hordeum vulgare* L.). If occurring early in the season, it has the ability to cause up to 30–40% yield loss in susceptible cultivars (Griffey 1994; Chaves 2008). The leaf rust development in barley is influenced by a complex interplay of factors, including environmental conditions such as those related to weather, field management practices, and the dominant pathotype, which can vary between years and locations (Clifford 1985). Although environmental conditions can strongly modulate epidemic dynamics, the genetic makeup of the host plant is a major determinant of resistance, and breeding for genetically resistant germplasm is a more economical, sustainable, and environment-friendly strategy for controlling leaf rust in barley than application of fungicides (Park 2008).

To date, at least 28 *Rph* (resistance to *P. hordei*) have been identified in barley (Park et al. 2015; Ziems et al. 2017; Kavanagh et al. 2017; Yu et al. 2018; Rothwell et al. 2020; Mehnaz et al. 2021; Clare 2024). These can broadly be classified into genes causing seedling/all-stage resistance (ASR) and genes conferring adult plant resistance (APR). ASR is typically race-specific and governed by major-effect genes that are relatively easy to identify; accordingly, most of the discovered *Rph* genes are ASR genes, but often rapidly overcome by evolving pathogen populations. In contrast, APR is generally not race-specific, controlled by multiple loci with smaller effects, and therefore more difficult for pathogens to overcome (Park 2015). Given their contrasting properties, breeding for both ASR and APR genes is considered an effective strategy for achieving both high levels of resistance and improved durability (Singh et al. 2025). For this reason, identifying new ASR and APR genes that confer resistance across diverse barley breeding populations and multiple environments is of great importance.

Approaches to identify genomic regions associated with leaf rust resistance in barley have involved genome-wide association studies (GWAS) based on diversity panels, mapping populations, or less frequently, breeding populations (Gutiérrez et al. 2015; Schnaithmann et al. 2014; Ziems et al. 2014, 2023; Vatter et al. 2018; Czembor et al. 2021; Kunze et al. 2024). Although some of these studies have been performed on panels including different growth types (spring versus winter) or row types (2-rowed versus 6-rowed) of barley, none of them have specifically focused on testing whether different types of barley harbor distinct quantitative trait loci (QTLs) for resistance. However, determining whether type-specific or common QTLs are involved is crucial, as it influences the transferability of findings between studies and supports our understanding of the genetic architecture of rust resistance in barley. While this remains an area of limited knowledge for rust resistance, previous studies have identified genetic variation for plant stature in different row-type and growth-type classes of barley (Alqudah et al. 2016). In addition, studies of hard red winter wheat and hard red spring wheat in the USA have shown that these two types typically differ in their leaf rust resistance genes, likely due to different pathogen races in different regions where spring and winter are grown (Kolmer 2007).

One way to compare the genetic architecture of a trait in different populations is to perform GWAS in each population separately and then compare the genome-wide presence and absence of peaks (Alqudah et al. 2016; Quero et al 2018). While this method is straightforward and simple, it does not leverage potential genetic correlations between populations. The absence of a common signal when comparing the independent GWAS results from two distinct populations could be due to several reasons including (1) insufficient statistical power due to small sample sizes within populations, (2) minor allele frequencies being too low to detect variant associations in either of the populations, (3) different patterns of linkage disequilibrium (LD) and/or linkage phases in the two populations, (4) different environmental factors affecting genotype-environment interactions in the different populations, and/or (5) different biological effects of causal variants between the studied populations. Further, the co-occurrence of a peak across two populations found using this method could still point to a case where the phase of the SNP and associated QTL allele is reversed between the populations due to recombination events, thus complicating transferability of knowledge from one population to another (Alqudah et al. 2016).

Another approach to explore common QTLs between populations is to take the opposite extreme and assume complete genetic correlation between subpopulations. Thus, the data from several populations would be combined into a univariate model estimating one effect per SNP. This approach has been applied to plants on several occasions (Alqudah et al. 2016; Müller et al. 2019; Zuffo et al. 2022). While the statistical power of this combined GWAS approach is greater than when populations are analyzed separately, due to a larger sample size, population-specific QTLs cannot be discovered, and the method can be highly biased if the sizes of the combined datasets are imbalanced and/or if allele frequencies in the populations are non-identical (Rio et al. 2020; Skovbjerg et al. 2025a).

A more statistically sound way to combine populations is to use a multivariate GWAS approach that jointly analyzes multiple populations by estimating population-specific SNP effects and their covariance. In this study, we extend the GWAS framework to enable systematic detection of common and context-specific SNP effects in applied barley breeding programs. Specifically, we use the framework to detect (1) environment-specific SNP effects within single populations of barley and (2) population-specific and common SNP effects across distinct barley breeding programs. While previous work has explored GWAS extensions for detecting SNP-by-environment interactions (Korte et al. 2012; Bornhofen et al. 2024), and other approaches have considered group-specific SNP effects across different heterotic groups in maize (Rio et al. 2020), the explicit testing of both population-specific and common SNP effects across more distinct populations (e.g., breeding programs with different breeding goals) remains underdeveloped.

## Materials and methods

### Plant materials

The dataset used in this study consisted of 6484 phenotyped inbred lines divided into four barley breeding programs: 581 lines belong to a 6-rowed winter (6RW) barley breeding program, 1640 lines belong to a 2-rowed winter (2RW) barley breeding program, 631 lines belong to a 6-rowed spring (6RS) barley breeding program, and 3632 lines belong to a 2-rowed spring (2RS) barley breeding program. All four programs were previously described in Skovbjerg et al. (2025a). While additional data have since been incorporated, their characteristics remain the same.

### Field trials and phenotyping

Field experiments were carried out from 2013 to 2024 depending on the population. For each year, genotypes were structured into trials that were located in Odder (OD), Denmark (55° 57′ 19.5″ N 10° 14′ 26.0″ E). In addition, the 2RW population were tested in Nienstädt (NI), Germany (52° 17′ 36.6″ N 9° 08′ 53.6″ E) in 2017–2018, and the 6RW population were tested in Güstrow (GU), Germany (53° 47′ 49.9″ N 12° 10′ 24.1″ E) in 2023. As susceptibility to leaf rust was only scored when the disease was present and sufficient resources were available, German locations are missing for most populations in most years. As a result, leaf rust susceptibility was assessed in 5 environments (combinations of year and location) for 6RW and 6RS, and in 10 and 11 environments for 2RW and 2RS, respectively. A specific combination of year and location is denoted as year/location (e.g., 2022/OD for year 2022, Odder).

Within an environment, genotypes were randomized using an alpha-lattice design with 2 replicates per genotype and a plot size of ∼1 m^2^. Field was managed according to the best management practices for conventional farming, except for fungicides which were not applied. Management practices were consistent across field locations, although herbicide applications were adapted to the weed pressure in the individual fields and to nationally approved products in Denmark and Germany, respectively.

Because phenotypes were routinely assessed in advanced breeding programs with new crosses generated each year, the set of lines varied annually, with partial overlap between years, especially between consecutive years (on average 10% of crosses proceed to the next year).

For each plot, leaf rust susceptibility was scored once or twice within an environment on a scale from 1 (resistant) to 9 (severely infected) as described in Skovbjerg et al. 2025b. Scoring was done at disease onset, and approximately 1 week later, reflecting BBCH stages 32 to 59 (node visibility to right before flowering; SEGES Innovation 2023). When two scores were available within an environment, they were averaged.

### SNP genotyping and filtration

DNA for SNP genotyping was extracted from two-week old seedlings as described in Skovbjerg et al. (2025a). All genotyping was done by TraitGenetics GmbH (Gatersleben, Germany). Individuals in the 2RS and 2RW populations were genotyped on two different SNP arrays depending on the year of analysis. Samples genotyped after 2013 were genotyped using the iSelect Illumina Infinium 15 K SNP chip, whereas those genotyped in 2013 or earlier were genotyped with a smaller iSelect Illumina Infinium 9 K SNP array. Prior to filtering, the dataset comprised 2513 6RW, 5951 2RW, 1349 6RS, 18,861 2RS individuals genotyped on the 15 K SNP chip, and 413 2RW and 1340 2RS genotyped on the 7 K SNP chip. This resulted in one VCF file per population and two VCF files per spring population. For each of the six VCF files, a SNP call rate filter of >0.8 was applied, and duplicate SNPs were removed. Missing genotypes were subsequently imputed separately for each population (6RW, 2RW, 6RS or 2RS) using Beagle v.5.4 (Browning et al. 2018, 2021) with default parameters and a sliding window of 150 cM. Because the 15 K SNP chip included all high-quality markers in the 9 K SNP chip, all genotype data were combined into one VCF file for imputation for 2RW and 2RS.

Following population-wise imputation, the four resulting VCF files were merged into a single genotype file, which was filtered to exclude SNPs with minor allele count (MAC) < 20 across populations (removed 3.8%). Further, samples lacking phenotypic data (78.7%), extreme population outliers as identified by principal component analysis (PCA) (< 1%), and near-identical genotypes (1.5%) (Skovbjerg et al. 2025b) were removed. The final dataset compromised 13,045 SNPs and 6484 individuals for downstream analyses.

### Genetic analyses of population structure, kinship, and linkage disequilibrium

Population structure was assessed using principal component analysis (PCA). This was done in R using the *prcomp* function on genotype data based on all 6484 plant lines and 13,045 filtered SNP markers (R Core Team 2013).

To investigate the relationships within populations, a block-wise genomic relationship matrix (*G* matrix) was computed, and relationship coefficients were plotted as a heatmap in R using the function *pheatmap* (R Core Team, R 2013). To correct for kinship and population structure in the GWAS, the **G** matrix was computed using the allele frequencies *across* populations by applying VanRaden’s method 2 (VanRaden 2008).

The number of private alleles per chromosome was counted within populations using the function *private_alleles* from the R-package *poppr* (v. 2.9.8, Kamvar et al. 2014; 2015).

Site frequency spectrums of different populations were made using a custom R-script which can be found at https://github.com/cks2903/RustResistancePaper/blob/main/FSFS_and_PrivateAlleles.

### Statistical models

All linear mixed models were applied using the DMU software package V. 6, release 5.6 (Madsen and Jensen 2013).

#### Analysis of variance

Variance partition performed on the phenotype data fitting the following linear mixed model:

1 $$\mathrm{y} ={\mathrm{X}}_{1}\mu + {\mathrm{X}}_{2}\mathrm{l}+{\mathrm{Z}}_{1}\mathrm{u}+{\mathrm{Z}}_{2}\mathrm{g}+{\mathrm{Z}}_{3}\mathrm{v} +\upvarepsilon$$

where **y** is a vector of observed phenotypes; µ is the fixed overall mean; **l** is a vector of the fixed effects associated with each unique year x location x trial combination; and **u** is a vector of random genomic breeding values for genotypes with $\mathbf{u} \sim N\left(0,\mathbf{G}{\sigma}_{u}^{2}\right)$ where ${\sigma}_{u}^{2}$ is the genomic variance captured by the additive SNP effects and **G** is the genomic relationship matrix; **g** is a vector of the random line effects not captured by the genomic breeding values with $\mathbf{g} \sim N\left(0,{\mathbf{I}\sigma }_{g}^{2}\right)$; **v** is a vector of the random interaction effect between year x location x line with $\mathbf{v} \sim N\left(0,{\mathbf{I}\sigma }_{w}^{2}\right)$; and ${\boldsymbol{\upvarepsilon}}$ is a vector of random residuals with ${\boldsymbol{\upvarepsilon}} \sim N\left(0,{\mathbf{I}\sigma }_{\varepsilon }^{2}\right)$. **X**_1_, **X**_2_, **Z**_1_, **Z**_2_, and **Z**_3_ are design matrices of fixed and random effects, respectively, and **I** denotes an identity matrix. To account for inbreeding, the reported values of ${\sigma}_{u}^{2}$ and *SE(*${\sigma}_{u}^{2})$ were multiplied by the average diagonal of the **G** matrix.

The broad sense design heritability (*H*^*2*^) and narrow sense design heritability (*h*^*2*^) were calculated on a line mean basis:

2 $$\widehat{{H}^{2}}= \frac{\widehat{{\upsigma}_{u}^{2}}+\widehat{{\upsigma}_{g}^{2}}}{\widehat{{\upsigma}_{p}^{2}}}$$

3 $$\widehat{{h}^{2}}= \frac{\widehat{{\upsigma}_{u}^{2}}}{\widehat{{\upsigma}_{p}^{2}}}$$

The phenotypic variance was calculated on an entry-mean basis as follows:

4 $$\widehat{{\sigma}_{p}^{2}}=\widehat{{\sigma}_{u}^{2}}+\widehat{{\sigma}_{g}^{2}}+\frac{\widehat{{\sigma}_{v}^{2}}}{{n}_{e}}+\widehat{\frac{{\sigma}_{\varepsilon }^{2}}{{n}_{r}}}$$

where *n*_*e*_ indicates the average number of environments per genotype, and *n*_*r*_ indicates the average number of observations per genotype across all locations.

#### Single-population GWAS

For GWAS within a single population, Eq. 1 was modified to include a SNP regression term (${\mathbf{m}}_{i}{\upbeta}_{i}$):

5 $$\mathrm{y} ={\mathbf{X}}_{1}\mu + {\mathbf{X}}_{2}\mathbf{l}+{{\mathbf{m}}_{i}\upbeta }_{i}+{\mathbf{Z}}_{1}\mathbf{u}+{\mathbf{Z}}_{2}\mathbf{g}+{\mathbf{Z}}_{3}\mathbf{v} +{\boldsymbol{\upvarepsilon}}$$

where all terms are as stated in Eq. 1, ${\upbeta}_{i}$ is the additive genetic effect of marker *i*, and **m**_*i*_ is a vector of genotypes (coded as 0,1, and 2) for marker *i*.

#### SNP × environment regression

All SNPs that were found to be statistically significant in single-population GWAS were tested for environment-specific effects using a SNP x E model. With this model, SNP regressions were nested within each unique combination of year and location. To avoid specific SNP x E interactions in picking up general g_a_xE interactions, which we know are present due to nesting of specific genotypes in specific environments, a gₐ × E term (${\mathbf{Z}}_{4k}\mathbf{w}$) was added as well resulting in the following model:

6 $${\mathbf{y}}_{k}={\mathbf{X}}_{1k}\mu + {\mathbf{X}}_{2k}\mathbf{l}+{{\mathbf{m}}_{ik}\upbeta }_{ik}+{\mathbf{Z}}_{1k}\mathbf{u}+{\mathbf{Z}}_{2k}\mathbf{g}+{\mathbf{Z}}_{3k}\mathbf{v}+{\mathbf{Z}}_{4k}\mathbf{w}+{{\boldsymbol{\upvarepsilon}}}_{k}$$

where all other terms are as stated in Eq. 5, with the exception of the environment-specific SNP effect ${\upbeta}_{ik}$ and genomic effects ${\mathbf{Z}}_{4k}\mathbf{w}$. In this model, breeding values consisted of main genomic effects $\mathbf{u} \sim N\left(0,\mathbf{G}{\sigma}_{u}^{2}\right)$ and environment-specific effects $\mathbf{w} \sim N\left(0,\mathbf{I}\otimes \mathbf{G}{\sigma}_{w}^{2}\right)$, where **I** is an identity matrix of with dimensions N_e_ x N_e_ where N_e_ is the number of environments.

To test for context-specific SNP effects, e.g., if SNP effects differed between two environments, all SNPs passing the significance threshold within a single-population GWAS was tested for different effects as follows:

7 $${\upbeta}_{\mathrm{D}\mathrm{E}\mathrm{T},\mathrm{i}}= \frac{{\widehat{\beta }}_{1,i}}{\mathrm{S}\mathrm{E}({\widehat{\beta }}_{1,i})}-\frac{{\widehat{\beta }}_{2,i}}{\mathrm{S}\mathrm{E}({\widehat{\beta }}_{2,i})}$$

where ${\upbeta}_{1,\mathrm{i}}$ and ${\upbeta}_{2,\mathrm{i}}$ are the additive genetic effects of marker *i* in environment 1 and 2, respectively.

The standard error associated with ${\upbeta}_{DET,i}$ was estimated as follows:

8 $${\mathrm{S}\mathrm{E}(\upbeta }_{\mathrm{D}\mathrm{E}\mathrm{T},\mathrm{i}})= \sqrt{{\mathbf{a}}_{\mathrm{D}\mathrm{E}\mathrm{T},\mathrm{i}}^{\mathrm{T}}{ \mathbf{V}}_{\mathrm{i}} {\mathbf{a}}_{\mathrm{D}\mathrm{E}\mathrm{T},\mathrm{i}}}$$

9 $${\mathrm{W}\mathrm{h}\mathrm{e}\mathrm{r}\mathrm{e} \mathbf{a}}_{\mathrm{D}\mathrm{E}\mathrm{T},\mathrm{i}}=\left(\begin{array}{c}\frac{1}{\mathrm{S}\mathrm{E}({\widehat{\beta }}_{1,i})}\\ -\frac{1}{\mathrm{S}\mathrm{E}({\widehat{\beta }}_{2,i})}\end{array}\right)$$

**V**_*i*_ denotes the variance–covariance matrix of the unscaled regressions for SNP *i*, and it has dimensions *n*_*p*_ x *n*_*p*_, where *n*_*p*_ is the number of compared groups included in the test.

A Wald test was used to associate ${\upbeta}_{\mathrm{D}\mathrm{E}\mathrm{T},\mathrm{i}}$ with a *p* value. To correct for multiple testing, a significance threshold of 0.05/n_t_ was applied, where n_t_ is the number of pairwise environmental comparisons performed for a given SNP.

#### Multi-population GWAS

For **multi-population GWAS** (*n* populations), Eq. 5 was expanded to reflect a multivariate model, where population-specific marker effects were estimated:

10 $$\left(\begin{array}{c}\begin{array}{c}{\mathbf{y}}_{pop1}\\ {\mathbf{y}}_{pop2}\\ \vdots \end{array}\\ {\mathbf{y}}_{popn}\end{array}\right)=\left(\begin{array}{c}\begin{array}{c}{\mathbf{X}}_{1,pop1}0 \cdots 0\\ 0 {\mathbf{X}}_{1,pop2}\dots 0\\ \vdots \vdots \ddots \vdots \end{array}\\ 0 0 \dots { \mathbf{X}}_{1, popn}\end{array}\right)\left(\begin{array}{c}\begin{array}{c}{\upmu}_{pop1}\\ {\mu}_{pop2}\\ \vdots \end{array}\\ {\mu}_{popn}\end{array}\right)+\left(\begin{array}{c}\begin{array}{c}{\mathbf{X}}_{2,pop1}0 \cdots 0\\ 0 {\mathbf{X}}_{2,pop2}\dots 0\\ \vdots \vdots \ddots \vdots \end{array}\\ 0 0 \dots { \mathbf{X}}_{2, popn}\end{array}\right)\left(\begin{array}{c}\begin{array}{c}{\mathbf{l}}_{pop1}\\ {\mathbf{l}}_{pop2}\\ \vdots \end{array}\\ {\mathbf{l}}_{popn}\end{array}\right)++\left(\begin{array}{c}\begin{array}{c}{\mathbf{m}}_{i,pop1}0 \cdots 0\\ 0 {\mathbf{m}}_{i,pop2}\dots 0\\ \vdots \vdots \ddots \vdots \end{array}\\ 0 0 \dots {\mathbf{m}}_{i,popn}\end{array}\right)\left(\begin{array}{c}\begin{array}{c}{\upbeta}_{pop1}\\ {\upbeta}_{pop2}\\ \vdots \end{array}\\ {\upbeta}_{popn}\end{array}\right)+\left(\begin{array}{c}\begin{array}{c}{\mathbf{Z}}_{1,pop1}0 \cdots 0\\ 0 {\mathbf{Z}}_{1,pop2}\dots 0\\ \vdots \vdots \ddots \vdots \end{array}\\ 0 0 \dots { \mathbf{Z}}_{1, popn}\end{array}\right)\left(\begin{array}{c}\begin{array}{c}{\mathbf{u}}_{pop1}\\ {\mathbf{u}}_{pop2}\\ \vdots \end{array}\\ {\mathbf{u}}_{popn}\end{array}\right)+\left(\begin{array}{c}\begin{array}{c}{\mathbf{Z}}_{2,pop1}0 \cdots 0\\ 0 {\mathbf{Z}}_{2,pop2}\dots 0\\ \vdots \vdots \ddots \vdots \end{array}\\ 0 0 \dots { \mathbf{Z}}_{2, popn}\end{array}\right)\left(\begin{array}{c}\begin{array}{c}{\mathbf{g}}_{pop1}\\ {\mathbf{g}}_{pop2}\\ \vdots \end{array}\\ {\mathbf{g}}_{popn}\end{array}\right)+\left(\begin{array}{c}\begin{array}{c}{\mathbf{Z}}_{3,pop1}0 \cdots 0\\ 0 {\mathbf{Z}}_{3,pop2}\dots 0\\ \vdots \vdots \ddots \vdots \end{array}\\ 0 0 \dots { \mathbf{Z}}_{3, popn}\end{array}\right)\left(\begin{array}{c}\begin{array}{c}{\mathbf{v}}_{pop1}\\ {\mathbf{v}}_{pop2}\\ \vdots \end{array}\\ {\mathbf{v}}_{popn}\end{array}\right)+\left(\begin{array}{c}\begin{array}{c}{{\boldsymbol{\upvarepsilon}}}_{pop1}\\ {{\boldsymbol{\upvarepsilon}}}_{pop2}\\ \vdots \end{array}\\ {{\boldsymbol{\upvarepsilon}}}_{popn}\end{array}\right)$$

All random effects, except the genomic breeding values (**u**), were assumed to be uncorrelated across populations.

In summary, our multi-population GWAS approach proposed three different tests (Table 1).

**Table 1:** Tests applied to test for significant marker-trait associations

Test name	Effect tested	Test type	H0
Average effect test (AET)	Average effect across populations	Wald test	$\mathrm{a}\mathrm{v}\mathrm{g}\left({\upbeta}_{1},{\upbeta}_{2}\right)=0$
Differential effect test (DET)	Different effects between populations	Wald test	${\upbeta}_{1}={\upbeta}_{2}$
Sign change test (SCT)	Cross-over interaction	Sign congruence test	${\upbeta}_{1}*{\upbeta}_{1}\ge 0$

**The average effect test (AET)** is worked by summing scaled SNP effects across two populations as follows:

11 $${\upbeta}_{\mathrm{A}\mathrm{E}\mathrm{T},\mathrm{i}}= \frac{{\widehat{\beta }}_{1,i}}{\mathrm{S}\mathrm{E}({\widehat{\beta }}_{1,i})}+\frac{{\widehat{\beta }}_{2,i}}{\mathrm{S}\mathrm{E}({\widehat{\beta }}_{2,i})}$$

where ${\upbeta}_{1,\mathrm{i}}$ and ${\upbeta}_{2,\mathrm{i}}$ are the additive genetic effects of marker *i* in populations 1 and 2, respectively.

The standard error associated with the common SNP effect was estimated as follows:

12 $${\mathrm{S}\mathrm{E}(\upbeta }_{\mathrm{A}\mathrm{E}\mathrm{T},\mathrm{i}})= \sqrt{{\mathbf{a}}_{\mathrm{A}\mathrm{E}\mathrm{T},\mathrm{i}}^{\mathrm{T}}{ \mathbf{V}}_{\mathrm{i}} {\mathbf{a}}_{\mathrm{A}\mathrm{E}\mathrm{T},\mathrm{i}}}$$

13 $${\mathrm{W}\mathrm{h}\mathrm{e}\mathrm{r}\mathrm{e} \mathbf{a}}_{\mathrm{A}\mathrm{E}\mathrm{T},\mathrm{i}}=\left(\begin{array}{c}\frac{1}{\mathrm{S}\mathrm{E}({\widehat{\beta }}_{1,i})}\\ \frac{1}{\mathrm{S}\mathrm{E}({\widehat{\beta }}_{2,i})}\end{array}\right)$$

and **V**_*i*_ is as described for the SNP x E test (Eq. 8).

For the **differential effect test (DET)**, the same algebra as used for SNP x E test was applied (Eqs. 7–9), where ${\upbeta}_{1,\mathrm{i}}$ and ${\upbeta}_{2,\mathrm{i}}$ now refer to the additive genetic effects of marker *i* in population 1 and population 2, respectively.

When applying DET to the average of populations 1 and 2 (group 1) and the average of populations 3 and 4 (group 2), the difference in group SNP effects was estimated as follows:

14 $${\upbeta}_{\mathrm{g}\mathrm{r}\mathrm{o}\mathrm{u}\mathrm{p} \mathrm{D}\mathrm{E}\mathrm{T},\mathrm{i}}= \frac{{\widehat{\beta }}_{pop1,group1,i}}{\mathrm{S}\mathrm{E}({\widehat{\beta }}_{pop1,group1,i})}+\frac{{\widehat{\beta }}_{pop2,group1,i}}{\mathrm{S}\mathrm{E}({\widehat{\beta }}_{pop2,group1,i})}-\frac{{\widehat{\beta }}_{pop3,group2,i}}{\mathrm{S}\mathrm{E}({\widehat{\beta }}_{pop3,group2,i})}-\frac{{\widehat{\beta }}_{pop4,group2,i}}{\mathrm{S}\mathrm{E}({\widehat{\beta }}_{pop4,group2,i})}$$

The standard error associated with the sum is as follows:

15 $${\mathrm{S}\mathrm{E}(\upbeta }_{\mathrm{g}\mathrm{r}\mathrm{o}\mathrm{u}\mathrm{p} \mathrm{D}\mathrm{E}\mathrm{T},\mathrm{i}})= \sqrt{{\mathbf{a}}_{\mathrm{g}\mathrm{r}\mathrm{o}\mathrm{u}\mathrm{p} \mathrm{D}\mathrm{E}\mathrm{T},\mathrm{i}}^{\mathrm{T}}{\mathbf{V}}_{\mathrm{i}} {\mathbf{a}}_{\mathrm{g}\mathrm{r}\mathrm{o}\mathrm{u}\mathrm{p} \mathrm{D}\mathrm{E}\mathrm{T},\mathrm{i}}}$$

The **a** vector used for estimating ${SE(\upbeta }_{\mathrm{g}\mathrm{r}\mathrm{o}\mathrm{u}\mathrm{p} \mathrm{D}\mathrm{E}\mathrm{T},i})$ is as follows:

16 $${\mathbf{a}}_{\mathrm{g}\mathrm{r}\mathrm{o}\mathrm{u}\mathrm{p} \mathrm{D}\mathrm{E}\mathrm{T},\mathrm{i}}=\left(\begin{array}{c}\begin{array}{c}\begin{array}{c}\frac{1}{\mathrm{S}\mathrm{E}({\widehat{\beta }}_{pop1,group1,i})}\\ \frac{1}{\mathrm{S}\mathrm{E}({\widehat{\beta }}_{pop2,group1,i})}\end{array}\\ -\frac{1}{\mathrm{S}\mathrm{E}({\widehat{\beta }}_{pop3,group2,i})}\end{array}\\ -\frac{1}{\mathrm{S}\mathrm{E}({\widehat{\beta }}_{pop4,group2,i})}\end{array}\right)$$

SNPs detected significantly by DET were subjected to a test for different signs of SNP effects using methodology described by Miller et al (2026). We labeled this test the **sign change test (SCT).**

Here, the null hypothesis of two SNP effects having the same sign (+ or −) was rejected if:

$${\widehat{\upbeta }}_{1}*{\widehat{\upbeta }}_{2}<0$$ and $$\mathrm{m}\mathrm{i}\mathrm{n}\left(\frac{\left|{\widehat{\upbeta }}_{1}\right|}{\mathrm{S}\mathrm{E}({\widehat{\upbeta }}_{1})},\frac{\left|{\widehat{\upbeta }}_{2}\right|}{\mathrm{S}\mathrm{E}({\widehat{\upbeta }}_{2})}\right)\ge {\Phi }^{-1}(1 -\upalpha )$$

where ${\Phi }^{-1}$ is the quantile function of a standard normal distribution, and α was liberally set to 0.05, since the test proposed by Miller et al. (2026) is already conservative for small *z*-values, as observed here.

No SNP x E testing was performed for the markers detected in multi-population GWAS due to its complexity.

#### Significance tests and proportion of variance explained by QTLs

Significance of SNP effects was tested by applying a Wald test to the *z*-values for SNP effects:

18 $$\mathrm{z}=\frac{\widehat{{\upbeta}_{\mathrm{i}}}}{\mathrm{S}\mathrm{E}({\widehat{\upbeta }}_{1})}$$

where β_i_ is the effect of SNP *i* as taken directly from Eq. 5 for single-population GWAS, or estimated using Eq. 7, 11 or 14 (depending on the scenario tested) for multiple-population GWAS. To account for multiple testing, variant associations were considered statistically significant if their *p* value were smaller than or equal to a Bonferroni threshold.

The proportion of additive genetic variance explained by SNPs (PVE) was calculated as described in Skovbjerg et al. (2025a):

19 $${\mathrm{P}\mathrm{V}\mathrm{E}}_{\mathrm{i}}=\frac{2*{\mathrm{p}}_{\mathrm{i}}*\left(1-{\mathrm{p}}_{\mathrm{i}}\right)*{\widehat{{\mathrm{b}}_{\mathrm{i}}}}^{2}}{\widehat{{\upsigma}_{\mathrm{g}\upalpha }^{2}}}$$

where ${\mathrm{p}}_{\mathrm{i}}$ is the allele frequency of SNP *i*, $\widehat{{\mathrm{b}}_{\mathrm{i}}}$ is the estimated regression coefficient of SNP *i*, and $\widehat{{\upsigma}_{\mathrm{g}\upalpha }^{2}}$ is the estimated additive genetic variance in the population before multiplication with the average diagonal element of **G**.

### Candidate gene search of GWAS hits

Candidate genes were identified using the *Hordeum vulgare* Morex V3 reference genome (Mascher et al. 2021). SNP markers were mapped to the same reference genome to obtain their genomic positions. A genome-wide gene table was constructed from the Morex V3 GFF3 annotation using a custom Python script. The script extracted key features including gene coordinates, strand orientation, gene length, the number of CDS exons, functional annotation, and confidence level. For each gene, the longest protein isoform was retained as the representative model. Candidate genes were identified by overlapping significant SNP regions with gene coordinates.

## Results

### Population structure and private alleles

**Fig. 1:**
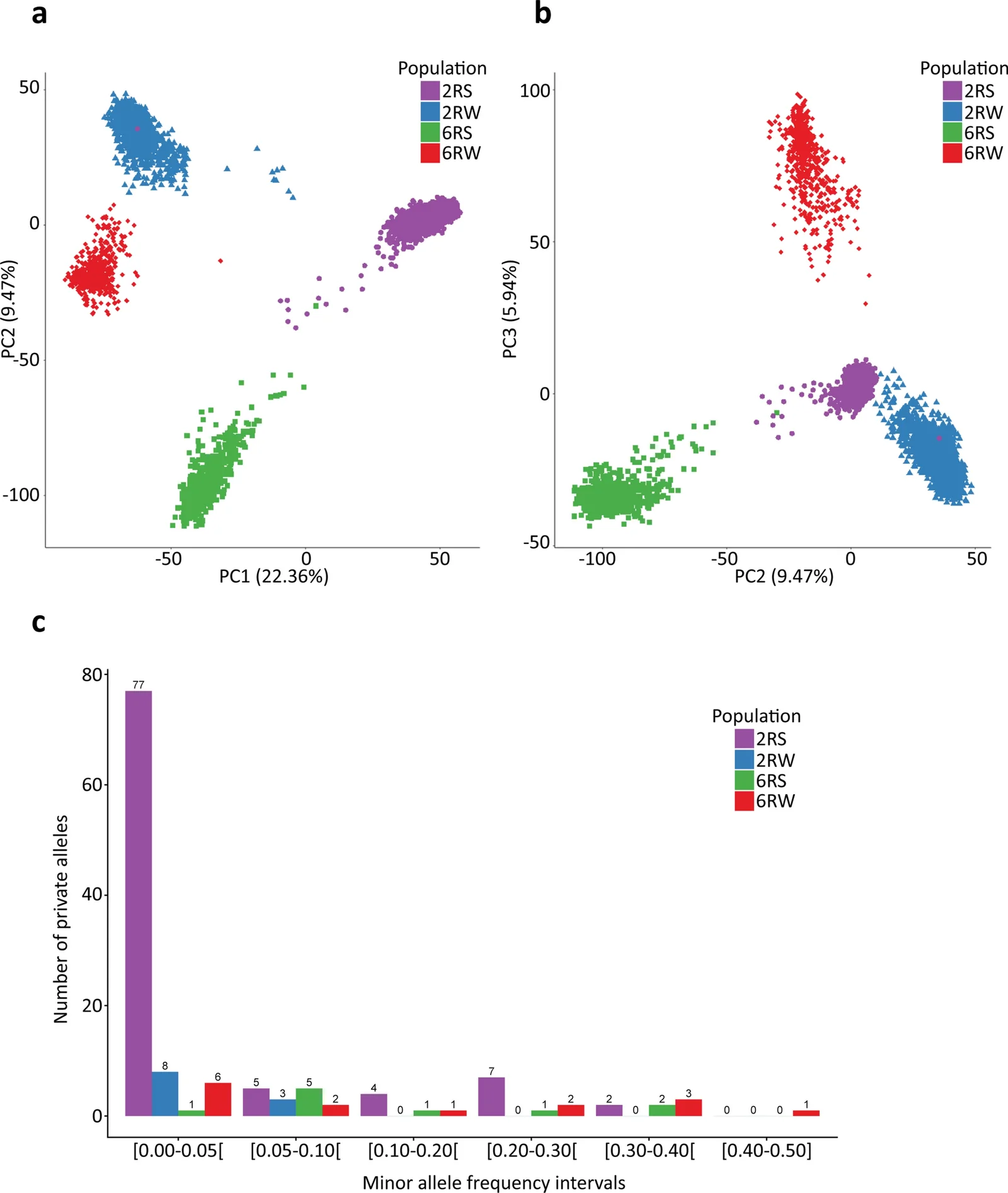
Population structure and private alleles in barley breeding populations. Genetic diversity among the four barley breeding populations by principal component analysis. (**a**) Scatterplot of the first two principal components (PC1 and PC2). (**b**) Scatterplot of the second and third principal components (PC2 and PC3). Each point represents an accession with population membership indicated by color and shape, and each axis states the proportion of genetic variance explained by the given PC (**c**) Number of private alleles identified within each population and their distribution across minor allele frequency (MAF) classes

To access the extent of population structure within and between populations, all 13,045 markers were used to perform a principal component analysis (PCA; Fig. 1a, b) and calculate a genomic relationship matrix (Fig. S1). Both analyses revealed that individuals were more genetically related within populations than between populations. Within populations, small blocks of close genetic relationships were observed, reflecting full-sib family structure. On average, low genomic relationship coefficients (6.9E-03) were found between populations, indicating genetic distinction between populations (Fig. S1). This distinction was confirmed by the PCA (Fig. 1a, b). The first three principal components (PCs) collectively explained 37.8% of the total genomic variance. In line with results on the same populations from Skovbjerg et al. (2025a), PC1 clearly separated winter and spring types, while PC2 tended to reflect row-type differentiation (Fig. 1a). In addition, PC3 fully separated the 6RW population from all others (Fig. 1b), indicating a unique genetic signature for this group.

Private alleles, defined as variants unique to a single population, can provide valuable information about population structure and gene flow between populations. However, they have limited applicability in multi-population GWAS, as the effect size of private alleles cannot be estimated across multiple populations. To evaluate the extent of this limitation in our dataset and further investigating population structure, we quantified the number of private alleles by population when grouped by population-specific minor allele frequency (MAF). For all populations, the number of private alleles was relatively small, ranging from 10 in the 6RS population to 95 in the 2RS population. As expected, the population with the largest sample size, 2RS, showed the largest number of private alleles, as well as the highest proportion of rare variants (MAF < 0.05) among those (Fig. 1c). Although larger sample sizes are expected to increase power to detect rare variants, the observed patterns were most likely a combination of larger sample size and extensive breeding of 2-rowed spring barley in Europe (Fischbeck 1992). Most private alleles in the 2RS population were located on chromosome 1H (Fig. S2), consistent with reported introgression at this chromosome driven by the incorporation of powdery mildew resistance at the *Mla* locus from wild and exotic germplasm into 2-rowed spring barley (Backes and Jahoor 2002).

While relatively few genetic variants were private to populations, site-frequency spectra revealed that the distribution of minor alleles was enriched for low frequency variants within all populations (Fig. S3). However, many variants that were rare within individual populations were also present in in other populations. To evaluate how the distribution of these rare variants changed when populations were combined, we identified variants with a MAC ≤ 10 in at least one population and recalculated their MAC after pooling individuals from all four populations (Fig. S4). The resulting distribution showed a substantial shift toward higher MAC values. Specifically, only 2.2% of the variants remained rare (MAC ≤ 10) after combining populations. This highlights the importance of combining populations in GWAS to increase allele frequencies and thereby improve the power to detect signals.

### Phenotypic variation and estimates of heritability

The distribution of the leaf rust susceptibility scores showed considerable variation within each population, both across different environments and within individual environments. Within each population, the most severe leaf rust infections occurred in different environments: 2022/OD for 6RW, 2015/OD for 2RW, and 2019/OD for 2RS. In contrast, the 6RS population showed relatively consistent infection levels across the recorded environments.

**Fig. 2:**
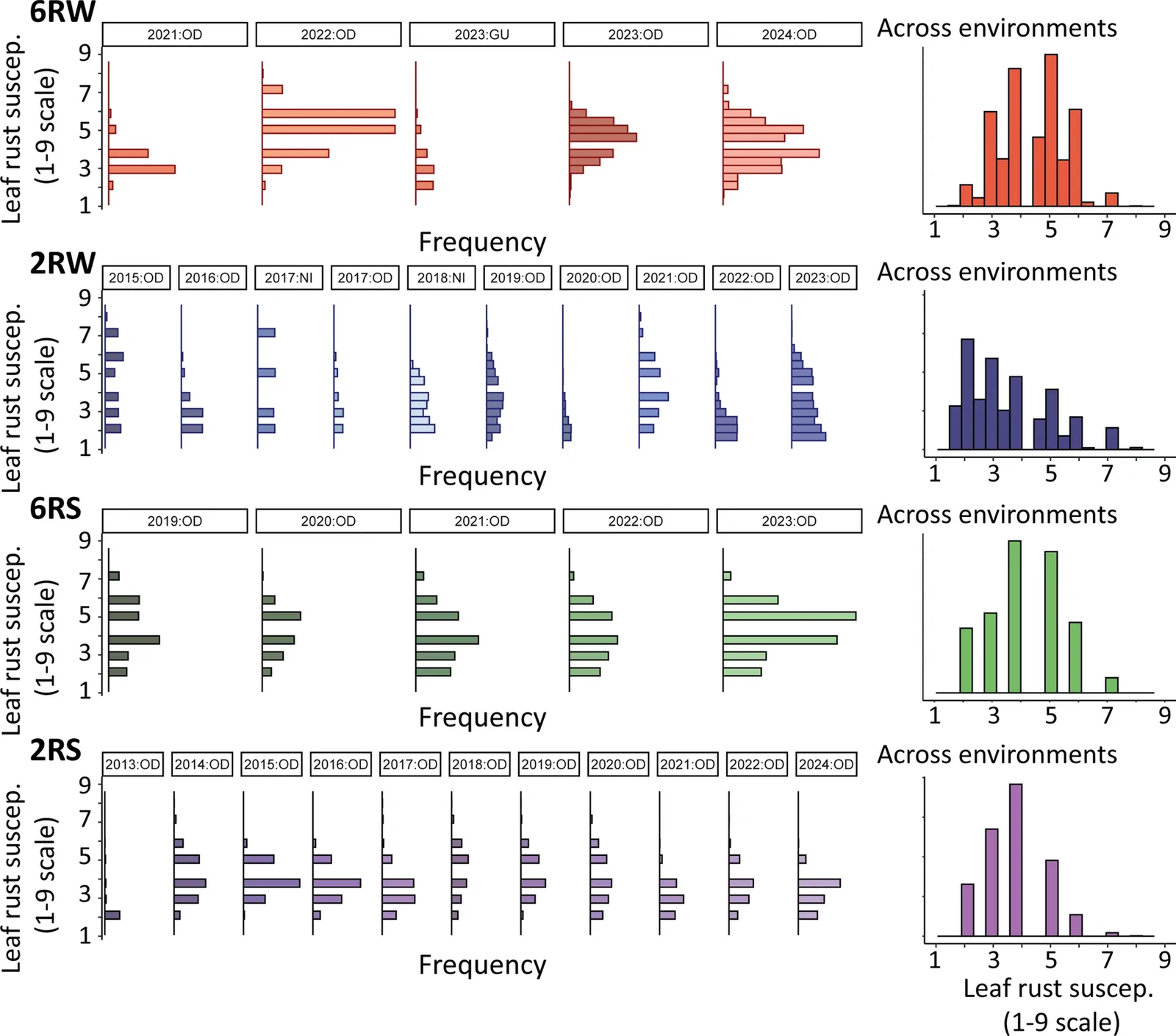
Trait measurements in different populations. Histograms of leaf rust scores within and across environments for each population

Across all environments, the median (50% quantile) leaf rust susceptibility score ranged from 3.0 in 2RW to 4.5 in 6RW. The 25% quantile for the 2RW population was notably lower than the other populations (1.5 versus 4.0–4.5), indicating that a substantial proportion of the 2RW lines might exhibit very little susceptibility (Fig. 2).

To investigate whether effects of genotypes on leaf rust scores vary across different environments, we calculated the Pearson correlation between phenotypic means of lines in pairwise combinations of years and locations. The estimated phenotypic correlations were moderate to high for most environments, indicating that phenotypic variance for rust resistance in our dataset is explained by GxE effects only to a smaller degree (Figs. S5–S8), as supported by the estimated variance components. Here, we found GxE to explain 6.9–23.4% of the overall plot variation within populations, whereas the main genetic effects (${\sigma}_{ga}^{2}$+${\sigma}_{gl}^{2}$) explained between 37.1 and 48.3% of the overall population-specific plot variation (Table 2). Encouraged by these results, we concentrated our GWAS on detecting QTLs with an effect on leaf rust resistance across all environments. To access the robustness of the genome-wide significant markers, we tested whether their effects differed between years, aiming to identify reliable and stable markers for breeding.

**Table 2:** Estimates of variance components and heritability within populations

Parameter	Population											
	6RW			2RW			6RS			2RS		
	Est	SE	%	Est	SE	%	Est	SE	%	Est	SE	%
Additive genetics variance (σ_ga_^2^)	4.8E−01	7.4E−02	48.3	7.7E−01	4.8E−02	41.9	1.04	1.64E−01	44.7	4.00E−01	3.90E−02	32.2
Line variance (σ_gl_^2^)	5.1E−06	2.3 E−02	0.0	7.0E−03	3.0E−02	0.4	3.35E−02	7.04E−02	1.4	6.05E−02	1.44E−02	4.9
Line effect x environment variance (σ_v_^2^)	7.4E−02	2.5E−02	7.4	4.3E−01	3.7E−02	23.4	3.62E−01	7.43E−02	15.6	8.62E−02	1.53E−02	6.9
Residual variance(σ_e_^2^)	4.4E−01	2.0E−02	44.3	6.3E−01	1.8E−02	34.2	8.86E−01	4.12E−02	38.2	6.96E−01	1.27E−02	56.0
Broad sense (*H*^*2*^)	0.72	0.04		0.59	0.03		0.64	0.05		0.62	0.02	
Narrow sense (*h*^*2*^)	0.72	0.04		0.58	0.03		0.62	0.05		0.54	0.03	

“%” columns indicate the percentage of overall variance explained by the parameter. *Est*. estimate; *SE* Standard error

### GWAS within populations

**Fig. 3:**
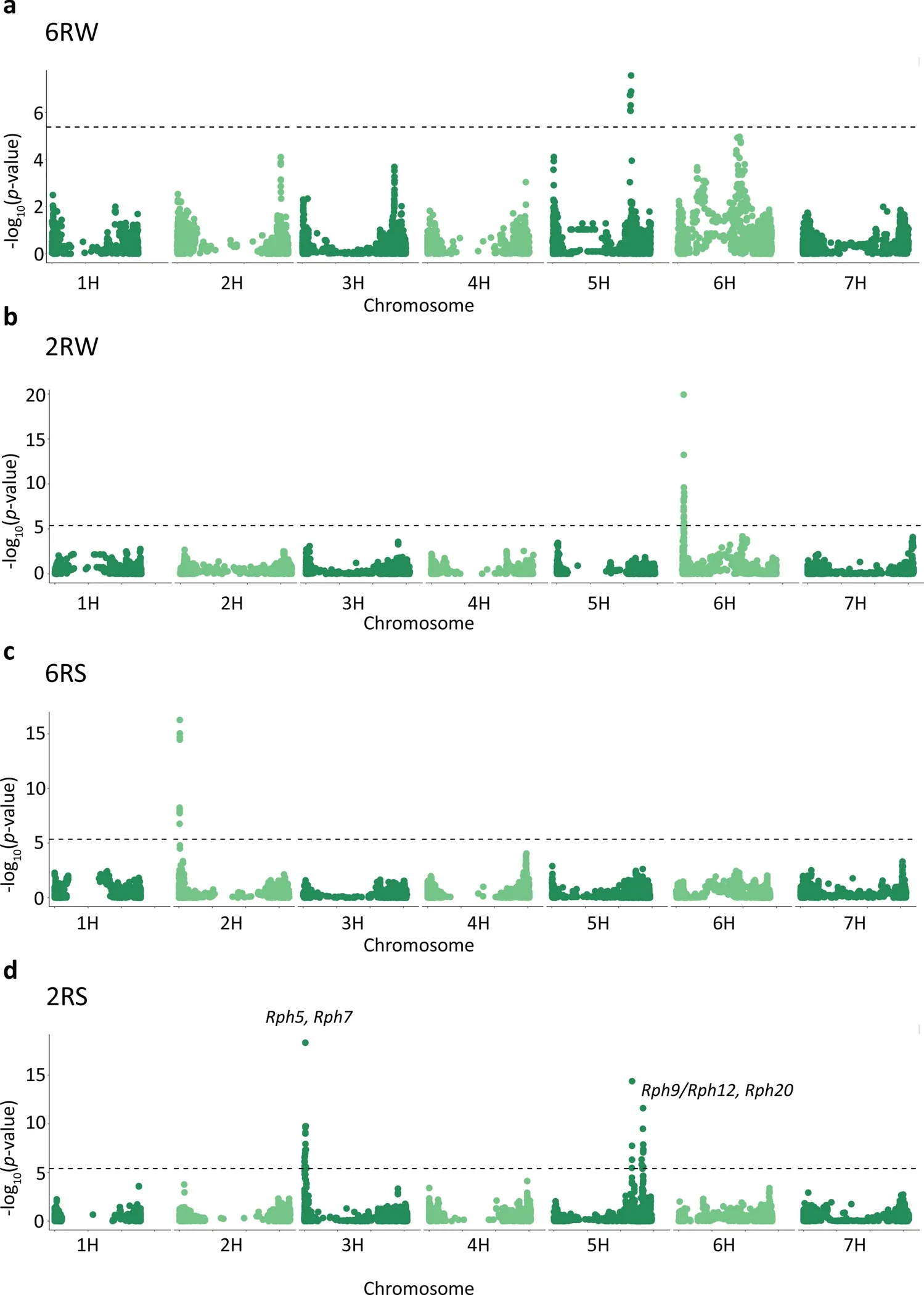
Single-population GWAS results. Manhattan plots for GWAS within (**a**) 6RW, (**b**) 2RW, (**c**) 6RS, and (**d**) 2RS. The x-axis displays the genomic position of markers by chromosome, and the y-axis displays -log_10_ of the GWAS *p* values. The dashed horizontal line reports the Bonferroni-corrected significance threshold. The genomic position of earlier described *Rph* genes colocalizing with detected signals is indicated with labels

Performing GWAS within each population detected 9 marker-trait associations (MTAs) for 6RW, 16 MTAs for 2RW, 9 MTAs for 6RS, and 38 MTAs for 2RS (Figs. 3, S9, Table S1). By considering physical proximity and LD between markers, MTAs were grouped into larger QTLs with each QTL being characterized by their lead SNP (Table 3). To better characterize the genomic basis of the detected signals, we explored the candidate genes within each GWAS-reported genomic region (Supplementary file 4). In 6RW, the significant associations corresponded to a single QTL on chromosome 5H at 463.3–470.4 Mbp, which explained 14.2% of the additive genetic variance. The genomic region contained 135 genes, and the lead SNP was annotated as a succinate–CoA ligase subunit beta and was located near a gene annotated as a disease resistance protein (TIR-NBS-LRR class, HORVU.MOREX.r3.5HG0488450).

**Table 3:** QTLs from single-population GWAS

Pop	Chr	Genomic region (bp)	Lead marker	*p*	MAC	PVE(%)	Annotation
6RW	5H	463,278,884–470,442,284	SNP9197	2.70E−08	47	14.2	Succinate–CoA ligase [ADP-forming] subunit beta
2RW	6H	8,185,797–12,209,320	SNP11460	1.16E−20	1420	46.2	Aspartate aminotransferase
6RS	2H	3,757,819–5,121,047	SNP4564	5.46E−17	423	35.3	Protein kinase
2RS	3H	1,668,436–12,322,905	SNP12434	4.63E−19	330	6.8	Glycine-rich domain-containing protein 2
2RS	5H	468,392,106–469,555,931	SNP12103	4.13E−15	429	5.9	Ribosomal RNA small subunit methyltransferase F
2RS	5H	527,203,338–537,215,281	SNP901	2.48E−12	1173	9.8	Signal transducing adapter molecule 2

*Chr* Chromosome, *MAC* minor allele count, *Pop* population, *PVE* proportion of additive genetic variance explained by the lead marker

In 2RW and 6RS, associations were likewise confined to single QTLs on chromosomes 6H (8.1 – 12.2 Mbp) and 2H (3.8–5.1 Mbp), respectively. These explained 46.2 and 35.3% of the additive genetic variance in 2RW and 6RS, respectively. The top marker of the 6RS peak resided within a protein kinase gene (HORVU.MOREX.r3.2HG0097640) that was in close proximity to three genes encoding NBS-LRR disease resistance genes. The top marker identified in 2RW was located within an annotated aspartate aminotransferase gene.

In contrast, 2RS revealed three distinct QTLs: one on chromosome 3H (1.7–12.3 Mbp) which explained 6.8% of the phenotypic variance and colocalized with the reported regions for *Rph*5 and *Rph7* (Chen et al. 2023; Clare et al. 2024) and two on chromosome 5H (468.4–469.6 Mbp and 527.2–537.2 Mbp) which explained 5.9 and 9.8% of the phenotypic variance, respectively. The first of the 2RS signals on chromosome 5H colocalized with the QTL detected in 6RW, and the second colocalized with the genomic position of *Rph20* (Hickey et al. 2011; Clare et al. 2024). In contrast to the 2RS population, we found no colocalization of known *Rph* genes and the regions reported in the 6RW, 2RW, and 6RS populations.

**Fig. 4:**
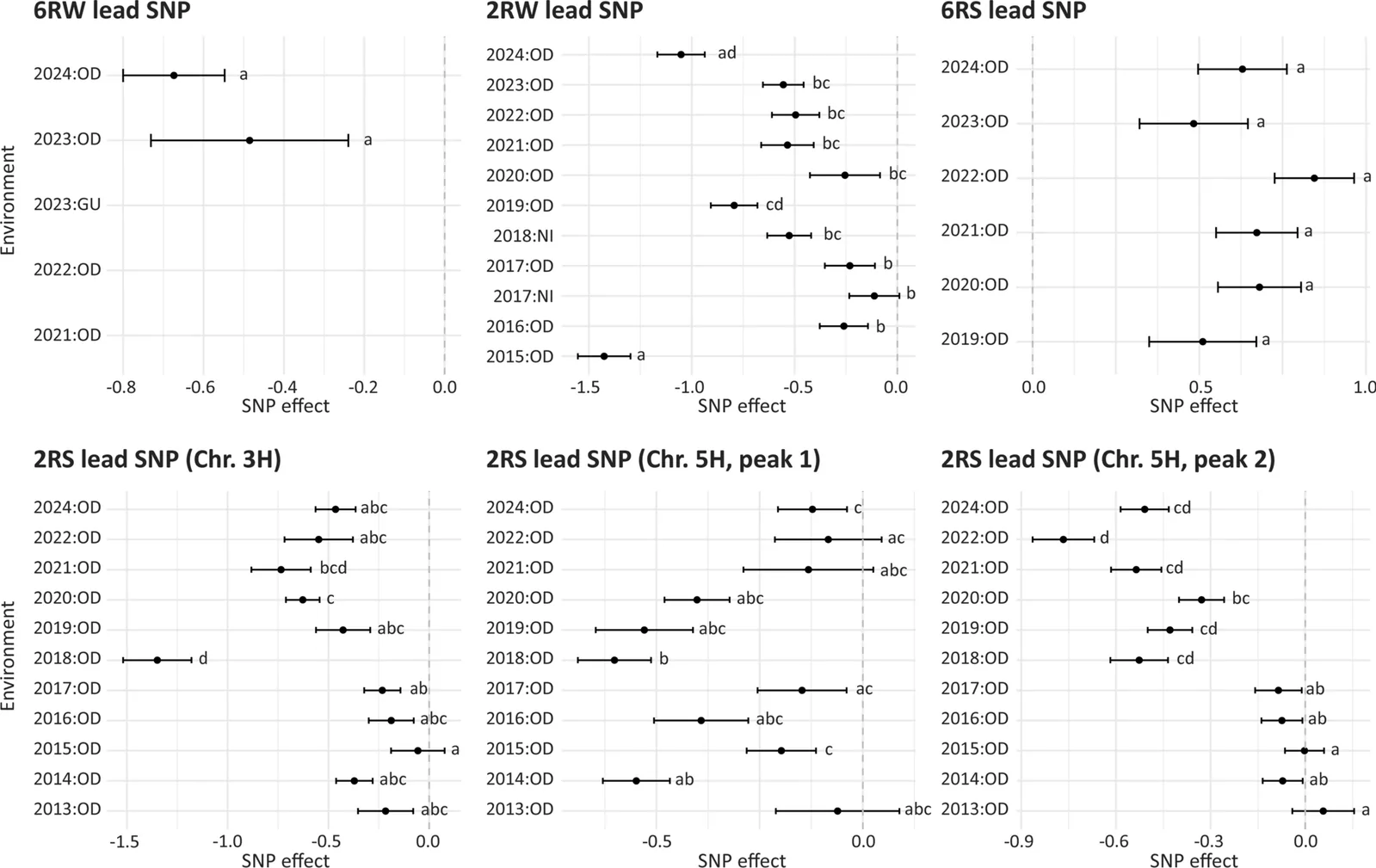
Environment-specific effects of lead SNPs identified in single-population GWAS. For each genomic signal within populations, the lead SNP effect was estimated within each environment. Point represents SNP effect estimates, and horizontal bars indicate their associated standard errors. The dashed vertical lines denote zero effect. Environments are labeled by year and location separated by “:”. Letters indicate statistically significant differences among environments of a given SNP within a population based on pairwise comparisons with a multi-testing correction a $\alpha \hspace{0.17em}$= 0.05

All the lead SNPs identified in single-population GWAS (Table 3) were tested for environment-specific SNP effects (Fig. 4). We found that effect sizes of a SNP varied significantly between environments, but that the direction of the effect (negative or positive) was consistent across environments for at least 5 out of 6 SNPs (Fig. 4). This suggests that while some markers might be more beneficial under a given set of environmental circumstances, it is unlikely that selection for those markers will harm the selection goals under another set of environmental circumstances. Nonetheless, we observed that for some markers, the magnitude of their effects varied remarkably across environments. For the lead SNP of the second peak on chromosome 5H in 2RS, the marker effect was close to zero during the years 2013–2017 but shifted to a significant effect of − 0.33 to − 0.77 units on the disease scale per copy of the minor allele during the years 2018–2024. In addition, we observed markers with relatively large effects in a few specific environments such as the 2RS lead SNP on chromosome 3H in 2018/OD and the 2RW lead SNP in 2015/OD (Fig. 4). While these sudden environment-specific effects of SNPs could be interpreted as a change of the biological or physical environments such as shifts in the dominating pathogen pathotype or weather conditions, it could also originate from changes in allele frequencies due to the nesting of genotypes in years or environments. For this reason, we investigated the environmental specific allele frequency for each of these six SNP x E marker effects (Fig. S10). Overall, we found that the changes in allele frequency did not reflect the changes in SNP effect magnitude.

### Common and population-specific GWAS hits

**Fig. 5:**
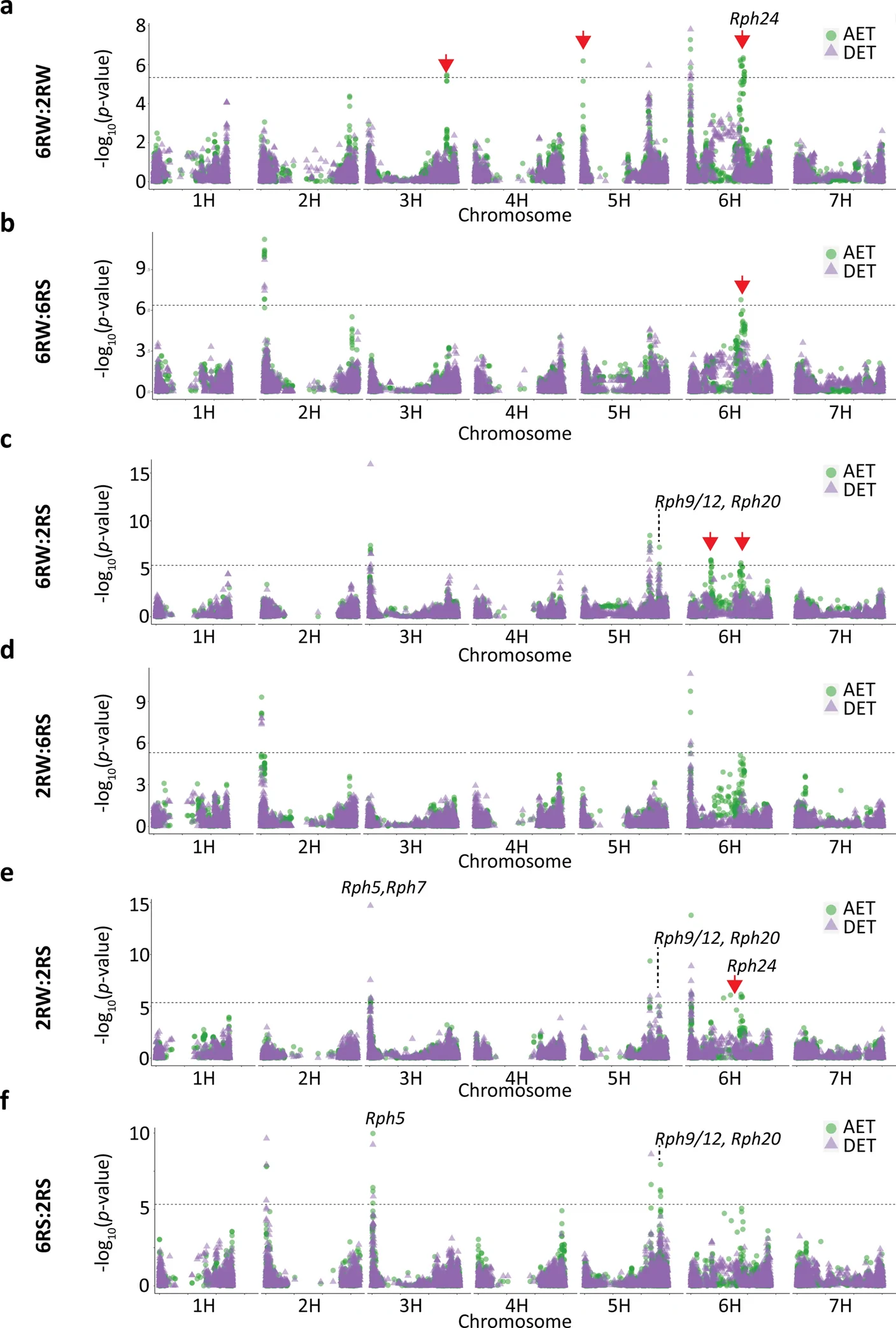
Pairwise multi-population GWAS results. Manhattan plots displaying results from multi-population GWAS on the following pairs of populations (**a**) 6RW:2RW, (**b**) 6RW:6RS, (**c**) 6RW:2RS, (**d**) 2RW:6RS, (**e**) 2RW:2RS, and (**f**) 6RS:2RS. The purple triangles display results from applying the differential effect test (DET), whereas the green circles display results from applying the average effect test (AET).The dashed horizontal line reports the Bonferroni-corrected significance threshold. The x-axis displays the genomic position of markers by chromosome, and the y-axis displays -log_10_ of the GWAS *p* values. The genomic position of earlier described *Rph* genes colocalizing with detected signals is indicated with labels. Red arrows point to QTLs that were not reported in single-population GWAS but were significant when applying the AET

To detect both common and population-specific genomic regions associated with leaf rust resistance among different types of barley, we performed pairwise multi-population GWAS of the four populations using the described AET, DET, and SCT tests (Figs. 5, S9, Table 4). We only considered markers with a MAC ≥ 10 within the combined populations to avoid identification of false positives (Table S2).

**Table 4:** QTLs from multi-population GWAS

Pops	Test	Chr	Genomic region (bp)	Lead marker	*p*	MAC	PVE(%)	Annotation
6RW:2RW	AET	3H	551,523,213–551,666,614	SNP3085	3.31E−06	541;665	8.7; 3.5	nuclease
	AET	5H	6,907,205	SNP3490	6.38E−07	238;145	6.4; 2.9	ADP-ribosylation factor
	DET,SCT	5H	467,301,344	SNP7621	1.10E−06	34;1055	8.9; 0.1	GRAM-containing/ABA-responsive protein
	AET	6H	8,185,797–9,839,762	SNP5003	5.23E−08	251;1328	6.8; 12.5	Ubiquitin-conjugating enzyme E2, putative
	DET	6H	9,079,863–12,209,320	SNP6460	1.53E−08	172;1306	<0.1; 16.5	NBS-LRR resistance-like protein
	AET	6H	356,868,076–386,177,094	SNP8401	4.41E−07	172;1306	14.2; 4.2	Helicase MOM1
6RW:6RS	AET	2H	3,757,819–5,789,931	SNP12258	4.35E−10	199;398	0.4; 33.2	Serine-rich 25 kDa antigen protein
	DET	2H	5,077,584–5,121,047	SNP4564	7.76E−09	228;423	<0.1; 34.4	Protein kinase
	AET	6H	356,868,076	SNP12236	2.23E−06	349;194	15.0; 6.1	Sister chromatid cohesion protein PDS5 B-B
6RW:2RS	AET	3H	5,281,870–6,260,961	SNP605	3.68E−08	106;934	0.8; 5.7	Glycosyltransferase
	DET,SCT	3H	6,032,885–9,182,522	SNP12434	1.23E−16	97;330	2.8; 9.2	Glycine-rich domain-containing protein 2
	AET	5H	467,301,344–469,057,208	SNP2617	3.32E−09	76;3359	3.3; 10.9	Homeobox protein, putative
	DET,SCT	5H	464,498,204–470,442,284	SNP9197	4.35E−08	47;52	14.1; <0.1	Succinate–CoA ligase [ADP-forming] subunit beta
	AET	5H	534,458,523–535,610,053	SNP901	5.65E−08	66;1173	0.2; 10.6	Signal transducing adapter molecule 2
	AET	6H	147,649,708–151,788,280	SNP9773	1.22E−06	238;23	7.9; 1.3	Outer envelope protein 80, chloroplastic
	AET	6H	358,462,574–358,462,594	SNP3939	2.41E−06	115;26	8.2; 0.8	RNA-binding family protein, putative
2RW:6RS	AET	2H	5,077,584–5,121,047	SNP12258	6.95E−09	1189;386	<0.1; 34.5	Serine-rich 25 kDa antigen protein
	DET	2H	5,077,584–5,121,047	SNP4564	1.54E−08	1195;423	0.1; 35.3	Protein kinase
	AET	6H	8,564,087–9,409,688	SNP11460	1.84E−10	1420;461	39.7; <0.1	Aspartate aminotransferase
	DET	6H	8,564,087–11,407,986	SNP11460	1.03E−11	1420;461	39.7; <0.1	Aspartate aminotransferase
2RW:2RS	DET,SCT	3H	5,281,870–9,182,522	SNP12434	1.99E−15	614;330	1.7; 9.2	Glycine-rich domain-containing protein 2
	AET	3H	6,032,885–8,850,348	SNP4643	1.64E−06	854;950	<0.1; 6.3	Glycosyltransferase
	AET	5H	468,392,106–469,555,931	SNP12103	4.27E−10	1285;429	0.3; 6.0	Ribosomal RNA small subunit methyltransferase F
	DET	5H	468,899,311–469,555,931	SNP12103	1.17E−06	1285;429	0.3; 6.0	Ribosomal RNA small subunit methyltransferase F
	DET,SCT	5H	527,206,555	SNP913	9.11E−07	884;317	2.2; 2.5	Protein kinase
	DET	6H	8,185,797–12,209,320	SNP6460	1.33E−09	1306;18	16.6; <0.1	NBS-LRR resistance-like protein
	AET	6H	8,564,087–10,100,689	SNP11460	1.68E−14	1420;1519	40.0; 0.2	Aspartate aminotransferase
	AET	6H	236,475,300–367,871,076	SNP12375	7.00E−07	168;24	4.7; 0.7	RNA-binding family protein, putative
6RS:2RS	DET	2H	3,758,616–5,121,047	SNP4564	2.27E−10	423;88	36.2; <0.1	Protein kinase
	AET	2H	5,088,355–5,121,047	SNP4564	1.44Ee−08	423;88	36.2; <0.1	Protein kinase
	AET	3H	6,032,885–6,260,961	SNP12434	1.07E−10	429;330	<0.1; 6.8	Glycine-rich domain-containing protein 2
	DET	3H	6,032,885–8,850,348	SNP12434	5.75E−10	429;330	<0.1; 6.8	Glycine-rich domain-containing protein 2
	AET	5H	469,555,931	SNP12103	2.34E−07	20;429	<0.1; 5.9	Ribosomal RNA small subunit methyltransferase F
	DET	5H	469,555,931	SNP12103	2.44E−09	20;429	<0.1; 5.9	Ribosomal RNA small subunit methyltransferase F
	AET	5H	534,458,523–536,730,207	SNP901	1.44E−08	31;1173	0.3; 9.8	Signal transducing adapter molecule 2
2R:6R	DET	5H	532,483,791	SNP3635	5.09E−06	107;2807	0.5; 0.2; <0.1; 3.8	Two-component response regulator

Minor allele count (MAC) values and percentage of additive genetic variance explained by the marker (PVE%) are reported for each population included in the analysis separated by a semicolon. The value before the semicolon refers to the first population listed, and the value after the semicolon refers to the second population listed. For example, the first row reports a MAC of 541 in 6RW and 665 in 2RW. For the PVE% for 2-rowed versus 6-rowed (2R:6R), the values are reported within each breeding population in the following order: 6RW, 2RW, 6RS, and 2RS. The annotation column reports the annotation of the gene that the reported top marker is located in. If the marker is intergenic, the annotation refers to the closest gene. Chr Chromosome, MAC minor allele count, Pops populations combined, PVE proportion of additive genetic variance explained by the lead marker

For all pairs of populations, the AET found 21 peaks with a significant average effect across populations (Fig. 5). Among these, 7 were unique to multi-population GWAS and therefore not found when the populations were analyzed individually (Fig. 5, red arrows). These included a region on chromosome 6H (356.87–386.18 Mbp for 6RW:2RW, and 236.48–367.87 Mbp for 2RW:2RS) which colocalized with the leaf rust resistance gene, *Rph24* (Fig. 5a–e). AET signals indicated common effects between populations, consistent with the significantly positive genetic correlations observed between most pairs of populations (Table S3).

The signals detected by the DET were less frequent and gave rise to 14 peaks with different effects between populations (Fig. 5, Table 4). Most of these (12/14) colocalized with AET signals, causing them to report markers where the average effect across populations is significant, while the effect differs remarkably in size across populations. Importantly, DET found 5 population-specific signals that pointed to marker effect with different signs across populations as evaluated by the SCT (Table 4). Considering the overlapping intervals reported by these peaks, they most likely constitute three QTLs.

Annotations for all genes located within the reported multi-population GWAS regions are provided in Supplementary file 5.

### Signals related to row-type specific rust resistance

**Fig. 6:**
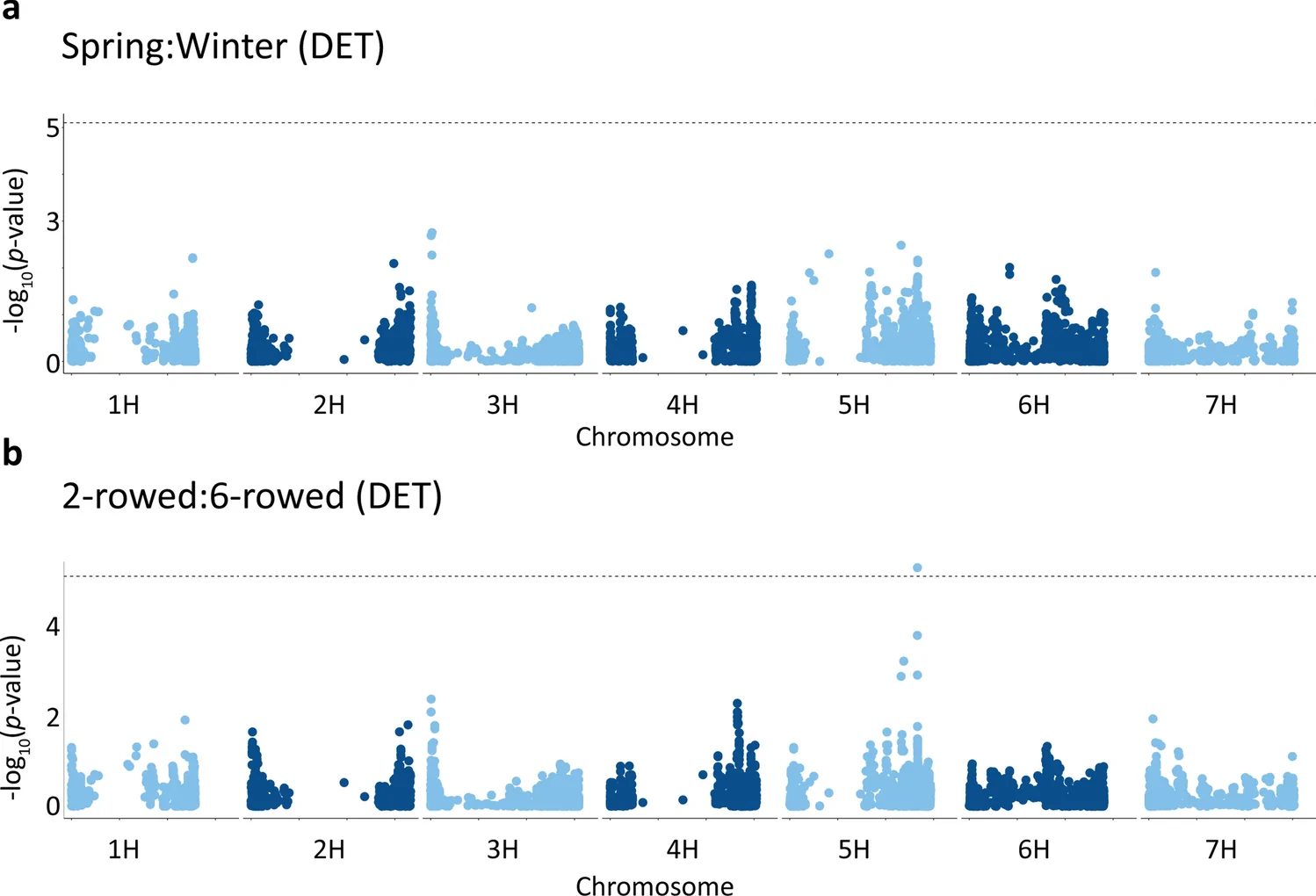
GWAS results for type-specific SNP effects. Manhattan plots displaying the results of applying the DET test to (**a**) different growth types of barley (spring versus winter) or (**b**) different row types of barley (2-rowed versus 6-rowed). The x-axis displays the genomic position of markers by chromosome, and the y-axis displays -log_10_ of the GWAS *p* values. The dashed horizontal line reports the Bonferroni-corrected significance threshold

Finally, we combined data from all four populations in a multivariate GWAS model using the DET that allowed us to test for markers statistically associated with leaf rust resistance that differed systematically in their effect of winter versus spring types or 2-rowed versus 6-rowed types (Fig. 6). Here, only m = 6391 SNPs passed our filter for MAC ≥ 10 including 60 of the 67 unique SNPs detected when combining populations pairwise (Table 4). A single SNP was revealed to differ significantly in effect size between the 6-rowed and 2-rowed populations (Fig. 6b). No signals were found to be associated with the separation of growth types (Fig. 6a).

## Discussion

### Single-population GWAS reveal major disease resistance genes

The simplest strategy for addressing genetic heterogeneity across populations in GWAS is to analyze each population separately. Using this approach, we detected a single peak in each population, except in the larger 2RS population where we observed 3 peaks. This indicates that leaf rust resistance is influenced by a few genes with large effects. This was especially the case in the 6RS and 2RW populations, where the signals explained 35.3% and 46.2% of the additive genetic variance, respectively. In 6RS, the major QTL maps to a narrow region on chromosome 2H (3.76–5.12 Mb; 42 genes). Although several *Rph* genes have been reported on chromosome 2H, including *Rph1* (Tuleen and McDaniel 1971; Dracatos et al. 2019), *Rph8* (Martin et al. 2020), *Rph14*-*Rph18* (Chicaiza 1996; Jin et al. 1996; Ivandic et al. 1998; Pickering et al. 1998, 2000; Kicherer et al. 2000; Perovic et al. 2004; Kopahnke et al. 2004; Weerasena et al. 2004; Golegaonkar et al. 2009; Derevnina et al. 2015; Martin et al. 2020), and *Rph22* (Qi et al. 1998; Marcel et al. 2007; Jafary et al. 2006; Liu et al. 2011; Johnston et al. 2013), the identified interval is physically distinct from all previously described *Rph* loci. The closest known gene, *Rph1*, is located 7–8 Mbp away, making it unlikely that this is the gene reported by the identified region. Notably, the top marker of the peak resides within a protein kinase gene in close proximity to three genes encoding NBS-LRR disease resistance genes. These genes represent plausible candidates underlying the QTL, as both protein kinases and NBS-LRR proteins are commonly involved in pathogen recognition and downstream defense activation (DeYoung and Innes 2006; Couto and Zipfel 2016). While the QTL identified in 2RW is located in a gene annotated as an aspartate aminotransferase, it is merely 5.9 kb away from another kinase gene, which may also represent a candidate gene for further investigation. Collectively, these findings identify the 2RW and 6RS regions as very interesting candidates for MAS.

While the signal detected in the 6RW population explained a considerably smaller fraction of the genetic variance compared to the mentioned signals in 2RW and 6RS, the top marker was located near an annotated disease resistance protein (TIR-NBS-LRR class). This gene may represent a candidate contributing to the observed association. In addition to the novel QTLs identified in this study, several GWAS hits overlapped with the following previously reported *Rph* genes: *Rph5* (Mammadov et al. 2003), *Rph7* (Chen et al. 2023), *Rph9/12* (Dracatos et al. 2014), and *Rph20* (Hickey et al. 2011) supporting the biological validity of the detected associations.

### Marker effects are environment-dependent but robust in direction

Mechanisms affecting rust resistance in barley are influenced by environmental factors, e.g., temperature, humidity, sowing distance, etc. (Department of Agroecology, Aarhus University and SEGES Innovation n.d.-a, n.d.-b). It is therefore not surprising that 4 of the 6 QTLs identified in single-population GWAS exhibited some degree of QTL x environment interaction in the larger populations (2RW and 2RS). The remaining two QTLs, identified in the smaller populations (6RW and 6RS), showed no detectable QTL x environment interactions, most likely due to limited sample size and the low number of environments tested. Consistent with this, previous studies have reported that larger sample sizes are required to obtain adequate power to uncover QTL x environment interactions compared to marginal SNP effects (Lanktree et al. 2009; Saïdou et al. 2014). While our study did not focus on detecting QTL x E effects, but rather on assessing the sensitivity of already detected SNP effects to environmental conditions, future studies could aim at identifying environmental-dependent effects by screening all markers and thereby uncover molecular mechanisms behind resistance. Previous studies have identified such markers to be relevant for disease resistance in barley (Zhao et al. 2012).

We found no marker effects with sign changes across environments. This contrasts with previous studies reporting both magnitude and sign changes (Li et al. 2003; Bornhofen 2024), likely because these studies compared more different environments across countries, whereas our study may lack the environmental contrasts needed to detect such sign changes.

Markers tended to show effects in certain environments while having no effect in other environments. This indicates that while markers identified through GWAS using data from multi-environmental field trials are robust in direction, they may remain environment-dependent. This dependency manifests in two ways: (1) Markers may gain or lose importance over time, and (2) single environments exhibit significantly larger SNP effects. An example of the first case was observed in the 2RS population for the second peak on chromosome 5H, where the top marker had no effect from 2013 to 2017 but became significant from 2018 to 2024. This shift may reflect changes in the pathogen dynamics. Temporal variation in virulence patterns has been documented for *Puccinia hordei* (Nazareno et al. 2023), while temporal shifts in pathotype frequencies have been reported for *Puccinia triticina,* the causal agent of leaf rust in wheat (Hovmøller et al. 2023). In contrast, weather variation is more likely to cause isolated outlier effects rather than consistent shifts. An example of the this was observed in the 2RS population, where the lead SNP on chromosome 3H had a remarkably large effect in the OD/18 environment (Odder 2018), which was characterized by high drought index during June and July (average of 9.8; The Danish Meteorological Institute 2026), suggesting that environmental conditions may have amplified the effect of this locus. While GWAS-derived markers may show environment-dependent effects due to their linkage to the causal variants rather the necessarily representing the causal variant itself, diagnostic markers developed from cloned resistance genes directly identify the functional resistance allele and therefore provide more stable targets for marker-assisted selection (MAS). However, the effectiveness of the underlying resistance gene may still depend on environmental conditions such as the pathogen population composition.

### Multi-population GWAS reveal common QTLs

Although few overlapping signals were observed between markers detected within individual populations, several common QTLs were detected through multi-population GWAS (Table 4). The detection of shared AET signals and positive genetic correlations between populations suggests that leaf rust resistance is, to a large extent, controlled by common genetic factors across populations. In particular, the AET proved to be effective in detecting common QTLs that did not appear in either of the two populations when performing GWAS individually. Among these, we identified broad genomic regions on chromosome 6H that colocalized with resistance gene *Rph24* (Ziems et al. 2017). The remaining additional loci detected only in the multi-population GWAS did not contain obvious new candidate disease resistance genes. While combining datasets results in higher statistical power to detect small effect QTLs (Korte et al. 2012; Skovbjerg et al. 2025a), it also pointed to genomic regions useful for breeding purposes across populations (Table 5, breeding category C1).

**Table 5:** Breeding conclusions of lead SNPs identified in pairwise multi-population GWAS

				SNP Effect					
Pops	Chr	Genomic region (bp)	Lead marker	pop1	pop2	AET	DET	SCT	Breeding category
6RW:2RW	3H	551,523,213–551,666,614	SNP3085	N.S	N.S	−	N.S	N.S	C1
	3H	6,907,205	SNP3490	N.S	N.S	+	N.S	N.S	C1
	5H	467,301,344	SNP7621	+	N.S	N.S	+	S	C2
	6H	8,185,797–9,839,762	SNP5003	N.S	−	−	N.S	N.S	C1
	6H	9,079,863–12,209,320	SNP6460	N.S	−	−	+	N.S	C3
	6H	356,868,076–386,177,094	SNP8401	N.S	N.S	+	N.S	N.S	C1
6RW:6RS	2H	3,757,819–5,789,931	SNP12258	N.S	−	−	+	N.S	C3
	2H	5,077,584–5,121,047	SNP4564	N.S	+	+	−	N.S	C3
	6H	356,868,076	SNP12236	N.S	N.S	−	N.S	N.S	C1
6RW:2RS	3H	5,281,870–6,260,961	SNP605	N.S	−	−	N.S	N.S	C1
	3H	6,032,885–9,182,522	SNP12434	N.S	−	N.S	+	S	C2
	5H	467,301,344–469,057,208	SNP2617	N.S	−	+	N.S	N.S	C1
	5H	464,498,204–470,442,284	SNP9197	−	N.S	N.S	−	S	C2
	5H	534,458,523–535,610,053	SNP901	N.S	−	−	N.S	N.S	C1
	6H	147,649,708–151,788,280	SNP9773	N.S	N.S	+	N.S	N.S	C1
	6H	358,462,574–358,462,594	SNP3939	N.S	N.S	+	N.S	N.S	C1
2RW:6RS	2H	5,077,584–5,121,047	SNP12258	N.S	−	−	+	N.S	C3
	2H	5,077,584–5,121,047	SNP4564	N.S	+	+	-	N.S	C3
	6H	8,564,087–9,409,688	SNP11460	−	N.S	−	-	N.S	C3
	6H	8,564,087–11,407,986	SNP11460	−	N.S	−	-	N.S	C3
2RW:2RS	3H	5,281,870–9,182,522	SNP12434	N.S	−	−	+	N.S	C3
	3H	6,032,885–8,850,348	SNP4643	N.S	−	−	N.S	N.S	C1
	5H	468,392,106–469,555,931	SNP12103	N.S	−	−	+	N.S	C3
	5H	468,899,311–469,555,931	SNP12103	N.S	−	−	+	N.S	C3
	5H	527,206,555	SNP913	N.S	−	N.S	+	S	C2
	6H	8,185,797–12,209,320	SNP6460	−	N.S	N.S	−	N.S	C3
	6H	8,564,087–10,100,689	SNP11460	−	N.S	−	−	N.S	C3
	6H	23,6475,300–36,787,1076	SNP12375	N.S	N.S	+	N.S	N.S	C1
6RS:2RS	2H	3,758,616–5,121,047	SNP4564	+	N.S	+	+	N.S	C3
	2H	5,088,355–5,121,047	SNP4564	+	N.S	+	+	N.S	C3
	3H	6,032,885–6,260,961	SNP12434	N.S	−	−	+	N.S	C3
	3H	6,032,885–8,850,348	SNP12434	N.S	−	−	+	N.S	C3
	5H	469,555,931	SNP12103	N.S	−	−	+	N.S:	C3
	5H	469,555,931	SNP12103	N.S	−	−	+	N.S	C3
	5H	534,458,523–536,730,207	SNP901	N.S	−	−	N.S	N.S	C1

Breeding category C1, Breed for the same marker allele across populations; Breeding category C2, Breed for different marker allele across populations; Breeding category C3, Breed within populations as marker is only relevant to one population. “+” and “– “ refer to the sign of the SNP effect and are only listed if the SNP effect tested significant. *Chr*. Chromosome, *N.S.* Non-significant, *S*. Significant, *SCT* Sign change test

### Population-specific QTLs arise from multiple underlying causes

In addition to common QTLs, the AET mostly detected loci where the average signal was driven by a large effect in one population while having a relatively small effect in the other population. Such QTLs could be identified, as they also reached significance under the DET, highlighting the value of applying both tests before interpreting results and making breeding decisions. In fact, 12 of 14 QTLs identified in the DET test were also detected in the AET test. This type of QTLs constitutes most of the multi-population GWAS detected signals (Table 5, breeding category C3). Population-specific SNP effects can reflect both differences in statistical power and true effect heterogeneity among populations (Veturi et al. 2019).

The population-specific QTLs deserving particular attention are the five showing population-specific allele associations and difference in direction of effects according to the SCT. Here, using marker effect information from one population to decide which allele to breed for in another population opposes the intended selection (Table 5, breeding category C2).

Population-specific QTLs can arise from several mechanisms. First, differences in LD between populations may cause the same causal variant to be tagged by different marker alleles. Second, the causal variant may differ in frequency (or even be absent in one population). Third, effects may depend on the genetic background (epistasis) (Rio et al 2020). A fourth possible mechanism is GxE interaction, where effects differ because the studied barley populations experience distinct environments especially through distinct growing seasons of winter vs. spring types. In 2020, Rio et al. showed that it was possible to distinguish effect size heterogeneity caused by differences in allele frequency and genetic background using admixed individuals. However, studies in humans have shown that effect sizes differ more between populations than can be explained by differences in allele frequency and LD, highlighting the relevance of different gene-by-gene and gene-by-environment interactions in different populations (Wang et al. 2020; Mathieson 2021).

For a breeding perspective relying on MAS, identifying the underlying cause of effect heterogeneity across populations may be important. LD-driven differences suggest imperfect marker resolution and may be resolved by identifying the causal variant, making effects transferable. In contrast, epistatic effects limit transferability across genetic backgrounds, particularly when effects differ in sign, but such markers can still be very useful within populations, if the correct allele is tracked in each population. In short, the breeder should stay away from markers depending on non-reproducible backgrounds. Thus, GxE-driven loci can still be useful if associated with reproducible location effects in contrast to year effects.

### Insights into row types and growth habits

Recent studies have performed GWAS for key traits in barley separately for spring and winter types, either with the argument that they are evaluated in different growing seasons or that they show pronounced diversity in population profiles (Kunze et al. 2024; Yuan et al. 2026). In contrast, other studies have combined genetically distinct row types of barley into a single GWAS while correcting for population structure (Gutiérrez et al. 2015; Amouzoune et al. 2024). Although this does not allow for detection of population-specific SNP effects, it can allow increased power to identify common SNPs for the trait of interest, especially if the populations are similar in size (Skovbjerg et al. 2025a). However, by combining heterogeneous populations in a multivariate GWAS framework, we were able to perform statistical tests for population-specific as well as common QTLs.

Disease resistance QTLs might colocalize with genes associated with heading or maturation date, or arise from pleiotropic effects, as escape through early development is a strategy to avoid fungal diseases (Gutiérrez et al. 2015). The four breeding populations studied here differ in developmental timing (Skovbjerg et al. 2025a), which introduces potential association between phenology and disease resistance. In this context, a key strength of our multi-population GWAS approach is its ability to detect markers whose allele frequencies differ significantly between populations, provided that they are segregating in each population (MAC ≥ 10 within each population). Such markers may lack sufficient within-population variation to be detected in single-population GWAS, while their effects can be eliminated when combined in a univariate GWAS correcting for population structure. However, an important limitation of our approach is that markers with low MACs within individual populations are excluded during filtering. This restricts the detection of loci that are close to fixation within populations, especially when comparing groups such as different row types or growth habits. This might explain why no signals appeared in the GWAS grouping growth types of barley, and only one marker reached significance in the GWAS grouping row types of barley. The marker associated with row type was in a gene annotated as a two-component response regulator.

### Proposed analytical pipeline for multi-population GWAS

**Fig. 7:**
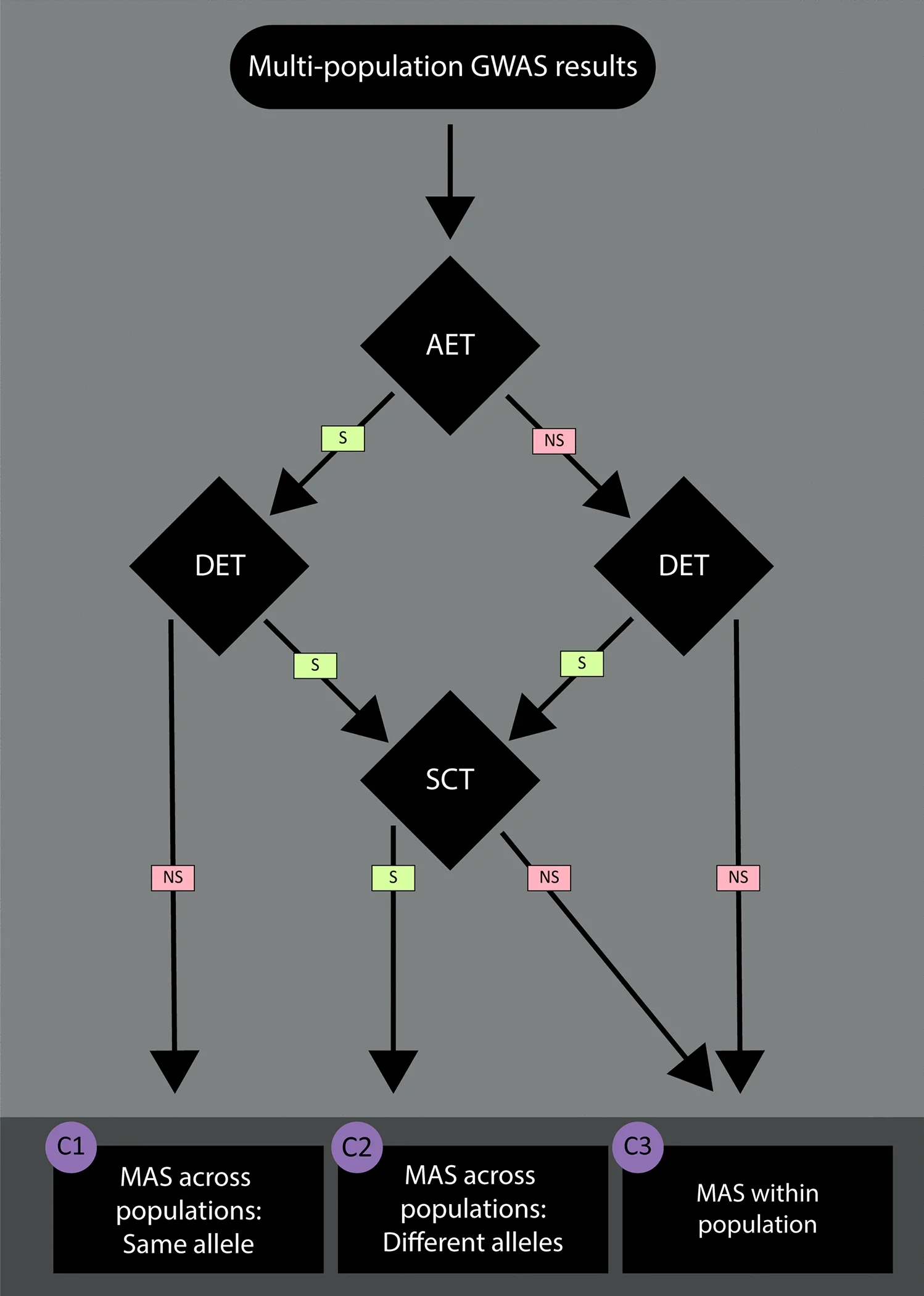
Suggested decision flowchart for multi-population GWAS hits. A flowchart to make breeding decisions based on the results of AET, DET and SCT. GWAS-significant SNPs are grouped into three categories C1-C3; Breeding category C1, Breed for the same marker allele across populations; Breeding category C2, Breed for different marker allele across populations; and Breeding category C3, Breed within populations as marker is only relevant to one population. Abbreviations: AET, average effect test; DET, differential effect test; MAS, Marker-assisted selection; N.S., Non-significant; S., Significant; and SCT, sign change test

Based on this study, we recommend multi-population GWAS to increase statistical power for detecting markers relevant across different populations. However, we also recommend that the resulting SNP effect be subjected to a set of tests (AET, DET, and SCT), and that results from all three tests be considered together before making breeding decisions. The proposed workflow is summarized in Fig. 7.

While common SNPs are valuable across populations, they should be interpreted with caution. They should be followed up by population-specific analysis on the difference in magnitude (DET) and direction (SCT) of effects between populations. Furthermore, the potential use of GWAS-identified QTLs in MAS depends on several factors, including effect size, stability across environments and populations, and the resolution of the associated genomic interval. Newly identified QTLs generally require further validation and fine-mapping before they can be converted into diagnostic markers for MAS.

## Supplementary Information

Below is the link to the electronic supplementary material.

Supplementary file 1 Genotype data (ZIP 1076243 kb)

Supplementary file 2 Phenotype data (CSV 1081 kb)

Supplementary file 3 (DOCX 2966 kb)

Supplementary file 4 A full list of all the genes located within the genomic regions corresponding to the signals identified in single-population GWAS (XLSX 122 kb)

Supplementary file 5 A full list of all the genes located within the genomic regions corresponding to the signals identified in multi-population GWAS (XLSX 331 kb)

Supplementary file 6 Table S1. All significant marker-trait associations identified in single-population GWAS (TXT 11 kb)

Supplementary file 7 Table S2. All significant marker-trait associations identified in multi-population GWAS (TXT 24 kb)

Supplementary file 8 Table S3. Estimated genetic correlations for leaf rust resistance between populations. All values are derived from the multivariate model including all four populations. In parentheses are standard errors (TXT 1 kb)

## Data Availability

All phenotype and genotype data used in this study can be found in supplementary files.
