# Supplementary material for: Discovering common and population-specific QTLs for leaf rust resistance in different Barley populations
Source: Theor Appl Genet. 2026 Sep 14;139(10):267. doi: 10.1007/s00122-026-05375-7 (PMC13574892; doi:10.1007/s00122-026-05375-7)
Supplement: Supplementary file 1 [file 122_2026_5375_MOESM3_ESM.docx]

**Supplementary Figures**

**Figure S1.**

**Figure S2.**

**Figure S3.**

**Figure S4.**

**Figure S5.**

**Figure S6.**

**Figure S7.**

**Figure S8.**

**Figure S9.**

**Figure S10.**
